# Streptococcus intermedius Bacteremia and Pyogenic Liver Abscess in a Patient With No Risk Factors

**DOI:** 10.7759/cureus.26786

**Published:** 2022-07-12

**Authors:** Sarah C Kurkowski, Michael J Thimmesch, Pinky Jha, Yasir H Abdelgadir

**Affiliations:** 1 Medical School, Medical College of Wisconsin, Milwaukee, USA; 2 Internal Medicine, Medical College of Wisconsin, Milwaukee, USA

**Keywords:** pyogenic liver abscess, extreme obesity, transdiaphragmatic extension, streptococcus intermedius bacteremia, pleural empyema, streptococcus intermedius

## Abstract

This case report depicts a 39-year-old male with no significant past medical history who was admitted for fever of unknown origin and sepsis. He was then found to have *Streptococcus intermedius* bacteremia and pyogenic liver abscess. The treatment course was complicated by pleural empyema leading to readmission. The case presented here adds to the medical literature, where a clear underrepresentation has been noted, and outlines a unique case of *S. intermedius* liver abscess complicated by pleural empyema in a patient without significant medical history, risk factors, or typical physical exam findings.

## Introduction

*Streptococcus intermedius* is an anaerobic, beta-hemolytic, gram-positive, non-motile, catalase-negative member of the *Streptococcus anginosus* group (SAG), also referred to as the *Streptococcus milleri *group. SAG is comprised of the following three species: *Streptococcus* *anginosus*, *Streptococcus* *intermedius*, and *Streptococcus constellatus*. The common characteristics among these three species are having a characteristic “caramel smell,” slow growth rate, ability to hydrolyze arginine, and inability to ferment sorbitol [1]. *S. intermedius* is a commensal organism found in the oral cavity and is a common component of dental plaque [2]. It is also found on other human mucosal surfaces (oral cavity, GI, and urogenital tract) and is part of the normal microbiota. Pathologically, *S. intermedius* is associated with liver abscess, bacteremia, brain abscess, osteoarticular infections, endocarditis, etc. [3,4].

A major pathogenic factor of *S. intermedius* is intermedilysin. Intermedilysin is potentially hemolytic to human red blood cells and results in human cell death with resultant membrane blebs. In the same study that brought light to the effect of intermedilysin, they demonstrated higher degrees of destruction dependent on which human cell type was exposed. Liver and brain cells were lysed to a higher degree than other human cell lines by intermedilysin [5]. This shed light on the reason why *S. intermedius* preferentially causes abscesses in the brain and liver parenchyma.

In addition, *S. intermedius* infection of specific organs has been linked to other characteristic virulence factors. Brain abscess and bronchopulmonary infections are associated with *S. intermedius* Sda histidine kinase, Listeria adhesion protein, and capsular polysaccharide biosynthesis protein cps4E. Liver abscess and abdominal abscess are associated with fibronectin-binding protein, fbp54, and capsular polysaccharide biosynthesis protein cap8D and cpsB [4]. Tissue damage is mediated by hyaluronidase (hydrolytic activity). Local inflammation is caused by proinflammatory cytokines. Hyaluronidase breaks down the hyaluronate component of connective tissue, providing the bacteria with nutrients. Fbp54 binds to fibronectin in the extracellular matrix, causing interleukin 8 (IL-8) to be released and recruiting neutrophils to the site of infection. However, neutrophils’ phagocytic properties are warded off by the *S. intermedius* polysaccharide capsule [3]. Intermedilysin binds to the human complement regulator CD59 (hCD59). It then destroys red blood cells through the creation of pores in the cell’s membrane. Another virulence factor specific to *S. intermedius* is sialidase A (NanA). NanA provides sialic acid molecules through the breakdown of sugar in the local area and on the bacteria surface. Sialic acid allows a communication between the bacteria and the host cell (such as a red blood cell) [3].

The first case of *Streptococcus milleri* group hepatic abscess was documented in 1975 [6]. Although it has been close to 50 years since the first documented case, a study published in 2020 (with a review of 101 recent case reports) stated that SAG species are still underrepresented in medical literature compared to other *Streptococci* species. They found that within the SAG, the least represented was *S. constellatus*, then *S. anginosus*, and finally *S. intermedius* [3].

There are multiple routes of bacterial seeding to the liver. The liver receives blood from the systemic and portal circulation. Therefore, compared to other organs, it is more at risk for bacterial infection from the biliary and gastrointestinal tracts. As stated, *S. intermedius* is a commensal organism of the oral cavity and thus can seed the liver hematogenously via the portal vein [4]. Systemic sepsis can seed the liver via the hepatic artery. In a study published in 2021, the most common origin was the arterial origin for pyogenic liver abscesses (PLAs) [7]. Infection in the biliary tract (ascending cholangitis) or peritoneal cavity can lead to liver abscess as well. The liver consists of four lobes; the right hepatic lobe is the most affected by PLAs due to its heavy vasculature. Up to 83-91% of PLAs are in the right lobe [8,9]. PLAs, without treatment, have been shown to have a mortality rate of 100%. However, treatment of the abscess decreases mortality to 2.5-14% [9].

The vast majority of patients with *S. intermedius* liver abscesses have pre-existing immunocompromised states such as diabetes, HIV, or drug use [10]. Other underlying conditions commonly present are pancreaticobiliary disease, malignancy, colon disorders, diabetes mellitus, hypertension, atrial fibrillation, and recent Whipple procedure or liver resection [6,11]. In one study, up to 28% had an unknown cause [11].

The case presented here adds to the medical literature, where a clear underrepresentation has been noted, as evidenced by our review of the literature and also other articles mentioned above [3], and outlines a unique case of *S. intermedius* liver abscess complicated by pleural empyema in a patient without significant medical history or risk factors.

## Case presentation

A 39-year-old male without any significant past medic It was recommended he present to the ED. In the ED, labs were notable for low hemoglobin and persistent history, aside from obesity with a BMI of 51.35 kg/m2, presented to the emergency department (ED) with weakness/fatigue, headache, cough, intermittent nausea, fever, and chills for two weeks duration.

Four days before presentation, the patient had seen his primary care team for complaints of fever, chills, sweats, cough, and nausea for nine days. He had completed multiple rapid coronavirus disease 2019 (COVID-19) tests, all of which were negative, and took ibuprofen without fever reduction. At the time, primary care considered viral illness the most likely diagnosis and prescribed albuterol for cough, ibuprofen for fever, and ondansetron for nausea. Primary care advised the patient to report to the ED if symptoms did not resolve in seven to 10 days. Prescription doses were ondansetron 4 mg 3x daily as needed and albuterol two puffs every four hours.

At the initial presentation to the ED, four days after the primary care visit, the patient reported minimal symptom improvement with ondansetron, Excedrin, ibuprofen, and Tylenol, along with chest pain. He denied shortness of breath (resolved since primary care visit), rhinorrhea, pharyngitis, vomiting, abdominal pain, rash, dysuria, hematuria, diarrhea, constipation, numbness, and tingling. Pertinent positives on the physical exam were tachycardia to 113 bpm and a fever of 101.9°F. Pertinent labs at presentation noted leukocytosis, hyponatremia, mild anemia, and mild elevation of lactic acid (Table 1). No significant wound was found on the skin exam. Chest X-ray showed no evidence of pneumonia; urinalysis showed no evidence of urinary tract infection. The patient had no travel history and no known sick contacts. Intervention in the ED consisted of 3 L of IV lactated Ringer’s solution and antibiotics (2 grams IV cefepime q8 hours and one dose of 100 mg oral doxycycline).

**Table 1:** Pertinent laboratory values at initial presentation

Lab	Value	Reference range
WBC	18.8 10^3^/mL	3.9-11.2 10^3^/mL
Hemoglobin	11.6 g/dL	13.7-17.5 g/dL
Lactic acid	2.2 (repeat 2.1) mmol/L	0.5-2.0 mmol/L
Na+	133 mmol/L	136-145 mmol/L
Total bilirubin	2.0 mg/dL	0.2-1.2 mg/dL
Direct bilirubin	1.3 mg/dL	0.3 mg/dL
C-reactive protein	17.96 mg/dL	<0.5 mg/dL
Erythrocyte sedimentation rate	94 mm/hr	0-25 mm/hr
Procalcitonin	7.39 ng/mL	<0.08 ng/mL
Platelets	702 10^3^/mL	165-366 10^3^/mL
Aspartate aminotransferase	90 U/L	13-44 U/L

The patient met systemic inflammatory response syndrome (SIRS) criteria for sepsis due to body temperature greater than 100.4°F, heart rate greater than 90 bpm, and leukocyte count greater than 12 x 10^3^/µL. He was admitted to internal medicine for the management of fever of unknown origin and sepsis.

The leading diagnosis at that time was presumed *Legionella* atypical pneumonia evidenced by fever, nonproductive cough, mild hyponatremia, leukocytosis, and unremarkable chest X-ray. A urine *Legionella* antigen test was ordered. CT of the chest was not done at that time as atypical pneumonia was suspected and the pulmonary physical exam was benign with no focal findings. The sepsis bundle management protocol was initiated. Blood cultures (x2) and respiratory cultures were drawn on hospital day one. Antibiotic treatment was switched to IV vancomycin (1500 mg IV q8 hours), cefepime (2 g IV q8 hours), and azithromycin (500 mg IV q24 hours). Additional labs noted elevated bilirubin, C-reactive protein (CRP), erythrocyte sedimentation rate (ESR), and procalcitonin (Table 1).

Infectious workup showed one of two blood cultures positive for gram-positive cocci in chains, identified as *S. intermedius*. Antibiotic sensitivities were determined by minimum inhibitory concentrations (MIC). IV ceftriaxone (2 g q24 hours) was continued, while vancomycin and azithromycin were stopped (Table 2). Continued infectious workup was negative for *Legionella*, *Streptococcus pneumoniae*, methicillin-resistant *Staphylococcus aureus*, *Staphylococcus aureus*, COVID-19, and influenza A/B. A transthoracic echocardiogram was completed due to the patient’s complaint of chest pain and to rule out any life-threatening cardiac involvement; no significant findings were noted and no concern for valvular diseases was present. Panorex did not show abscess or source of infection either.

**Table 2:** Minimum inhibitor concentration (MIC) of antibiotics against Streptococcus intermedius

Antibiotic	MIC	Sensitivity
Ceftriaxone	0.063 mg/mL	Sensitive
Penicillin	0.063 mg/mL	Sensitive
Vancomycin	1.000 mg/mL	Sensitive

**Figure 1:**
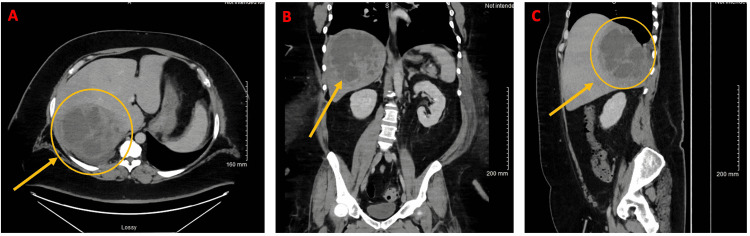
CT of the abdomen/pelvis with contrast on hospital day five (first admission) (A) Axial view of liver abscess. (B) Coronal view. (C) Sagittal view.

On hospital day five, CT of the abdomen/pelvis with contrast demonstrated a multiloculated/septated lesion that occupied the posterior right lobe of the liver. This was most consistent with an intrahepatic abscess measuring 15 cm in diameter. Also noted were findings in the right portal vein, right hepatic vein, and middle hepatic vein concerning venous thrombosis or compression due to mass effect of abscess, small right pleural effusion with atelectasis of right lower lobe, and sigmoid colon diverticulosis (Figure 1). Subsequently, interventional radiology performed ultrasound- and CT-guided drain placement into the right hepatic abscess. The 14 x 35 cm drain yielded 100 cc of thick purulent fluid and the drain was left in place. Abscess culture showed 4+ polymorphonuclear neutrophils (PMNs) and 4+ gram-positive cocci (GPC) with 3+ *S. intermedius*.

Liver/spleen Doppler ultrasound was negative for portal venous thrombus, as that was the concern noted on CT previously. The patient was discharged with a peripherally inserted central catheter (PICC) to complete a 14-day course of ceftriaxone (2 g IV q24 hours) as an outpatient.

**Figure 2:**
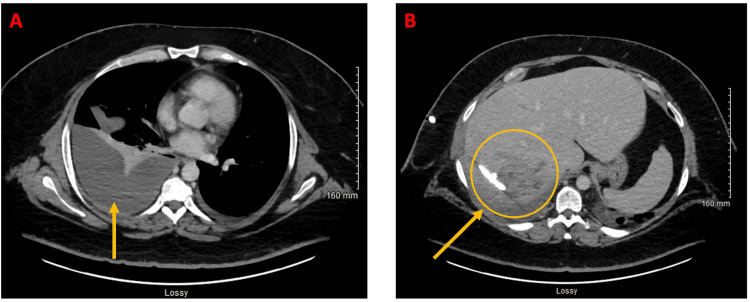
CT of the abdomen/pelvis with contrast on hospital day one (second admission) (A) Axial view of pleural effusion/developing empyema. (B) Axial view of resolving liver abscess with drain.

**Figure 3:**
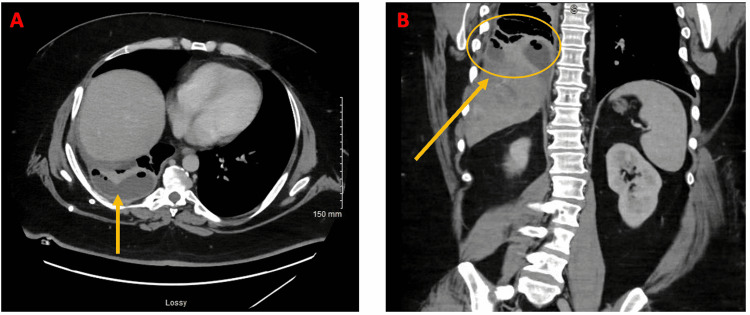
CT of the abdomen/pelvis with contrast on hospital day eight (second admission) (A) Axial view of loculations in empyema. (B) Coronal view of loculations in empyema.

Two days later, the patient called the nurse triage line because of intermittent profuse sweating and weakness. He was recommended to present to the ED. In the ED, labs were notable for low hemoglobin and persistent leukocytosis consistent with the known infectious process and were improved from the prior examination (Table 3). CT of the abdomen/pelvis with contrast was obtained and was concerning for pleural extension of the patient's known liver abscess. The case was discussed with infectious disease and cardiothoracic surgery teams. Cardiothoracic surgery recommended additional CT imaging for planning placement of a pleural drain. Subsequent CT of the abdomen/pelvis with contrast showed enlarging right pleural effusion and improving right hepatic abscess (Figure 2). Abscess size decreased from 15 cm (at largest diameter) to 4.9 x 6.9 x 8.5 cm. It was believed the right pleural effusion was likely an extension of liver abscess; however, it was not confirmed if it was an empyema with certainty. Ultrasound- and CT-guided right-sided thoracentesis without chest tube placement was performed, with 20 cc of pleural fluid sent for laboratory analysis. Pleural fluid was found to be exudative (Table 4). Culture from pleural fluid continued to have no growth to date (NGTD); however, this was expected, as *S. intermedius* is very sensitive to antibiotics (ceftriaxone). Though the presence of the exudative pleural fluid was concerning for complicated effusion (possibly bacterial) that would need further drainage. Interventional radiology placed a 12 French pigtail chest tube with a return of 40 mL of clear yellow fluid. The pleural collection had fine webs and loculations that were mechanically disrupted to increase fluid return during the procedure (these loculations/webs were below the resolution of imaging, thus not seen on CT of the abdomen/pelvis). Four days later, on hospital (second admission) day eight, CT of the abdomen/pelvis showed improving right hepatic abscess but interval loculation of right pleural effusion, likely conversion into empyema (Figure 3). IV ceftriaxone via PICC line was extended due to empyema. Due to concern for fistula between right hepatic abscess and right pleural cavity, a fluoroscopic diagnostic sinogram was performed. Linear tracks were seen medially and spreading cephalad, which was suspicious for fistula, but no definite connecting fistula was identified, though it could not be completely ruled out. Two days later, the patient was discharged with a PICC line for IV ceftriaxone for 14 days.

**Table 3:** Pertinent laboratory values at readmission

Lab	Value	Reference range
WBC	12.8 10^3^/mL	3.9-11.2 10^3^/mL
Hemoglobin	8.3 g/dL	13.7-17.5 g/dL
Platelets	633 10^3^/mL	165-366 10^3^/mL
Aspartate aminotransferase	62 U/L	13-44 U/L

**Table 4:** Pleural fluid lab values

Lab	Value
Glucose	4 mg/dL
WBC	317 (neutrophil predominant)
Pleural/serum protein ratio	>0.5
Lactate dehydrogenase	1835 U/L

Fourteen days post-discharge, the patient was seen in the infectious disease clinic for PICC line removal and the radiology clinic for right upper quadrant (RUQ) hepatic drain removal. The patient's IV ceftriaxone course was of 34 days; RUQ hepatic drain was in place for 30 days; chest drain was in place for six days. The first admission was for nine days and the second admission was for 11 days, leading to a total of 20 days in the hospital. The patient continued to be followed as an outpatient by infectious diseases and continued to show improvement of hepatic abscess and pleural empyema on symptoms, physical exam, and imaging.

## Discussion

This case report details a *Streptococcus intermedius* PLA with possible secondary pleural empyema in an unlikely patient with no significant medical history or commonly documented risk factors.

In general, without treatment, PLAs have a mortality rate of 100%. Treatment can reduce this percentage down to 2.5-14% [9]. Hepatic abscesses can occur via bile ducts, vessels, or direct inoculation by close structures. In the western world, bacterial (or pyogenic) liver abscesses are the most common (80% of PLAs), and mortality is close to 15% even with treatment. The high mortality has been hypothesized to be due to delays in management, comorbidities complicating disease course, and concomitant biliary disease [10]. This diagnosis is much more common in males versus females (ratio of 4:1) and in Caucasian individuals (up to 80% of cases) [11].

Multiple complications can arise from PLAs, with around 40% of patients experiencing a complication, including sepsis/septic shock, pleural effusion, abscess rupture, venous thrombosis, thoracic empyema, and mediastinitis [3,9,11]. The least common is thoracic empyema or mediastinitis, secondary to a communicating fistula [12]. Our patient’s case depicts the rare complication of empyema secondary to a PLA, likely due to communicating fistula between abscess and pleural cavity. Although unable to definitely diagnose a communicating fistula, the fluoroscopic diagnostic sinogram did provide additional support for it.

One article described the factors for infection with *S. intermedius*, stating that the top six risk factors in the study’s cohort were previous dental manipulation (18.8%), sinusitis (11.9%), diabetes mellitus (7.9%), heavy alcohol use (7.9%), congenital/acquired heart disease (7.9%), and malignancy (6.9%). A separate study from 2016 showed the main risk factors being solid tumors (32.1%) and diabetes mellitus (18%) in the cohort studied [3]. Many patients with *S. intermedius* PLA are found to be immunocompromised in some way, such as HIV, immunosuppressant use, or drug use [10]. What is significantly noteworthy about our case report is that the patient did not have any of the associated conditions listed above. The commonalities found in the literature review, and the lack of their presence in our patient, make this case a rare and atypical presentation.

The majority of PLAs (not solely those caused by *S. intermedius*) are located within the right hepatic lobe. One study specifically found 72.7% of abscesses to be in the right liver lobe [7]. Another study, completed in 2012, which reviewed 400 patients with PLA, reported that the right hepatic lobe was most frequently affected (83% of cases). In most cases, no identifiable cause could be found (cryptogenic in origin, 56%). Of identifiable cases, biliary tract disease was the most frequent cause of PLAs overall (44%). Other origins of infection were via the portal or arterial circulation (15% and 13.5%, respectively). Only 4.5% of PLAs affected solely the left hepatic lobe, whereas 12.5% affected both the right and left lobes. In a cohort studied, 21% of patients had multiple abscesses, and 50% had a single abscess at initial presentation. The most commonly responsible organisms were *Klebsiella* (45%) and *Escherichia coli* (32%) [8]. Our patient differed as neither of these organisms was the culprit of his PLA, and no biliary disease was found.

Our patient’s presentation was not the classical presentation for PLA or specifically abscess due to *S. intermedius*. In a 12-year retrospective study published in 2021 on 1572 patients with PLA, the most common clinical signs were fever (88.9%) and abdominal pain (51.3%). In the prospective case series published in 2012, with a cohort of 400 patients, it was found that the male to female ratio was 4:1. Most common presenting symptoms were as follows: upper abdominal pain (97%), RUQ tenderness (95%), high-grade fever (74%), nausea/vomiting (50%), loss of appetite (49.5%), localized guarding (47%), hepatomegaly (26%), and toxemic initial presentation (4.5%). Our patient presented with a fever of 101.9°F (not high-grade fever) and sepsis meeting SIRS criteria. However, he did not have RUQ tenderness, abdominal pain, nausea/vomiting, localized guarding, loss of appetite, etc., all of which are shown to be a significant portion of presenting symptoms. This further supports the claim that this case report is unique compared to previous literature on *S. intermedius* liver abscesses.

Imaging is the main way to diagnose a liver abscess, particularly through ultrasound or CT scan. Needle aspiration confirms the diagnosis and aids in antibiotic choice, as laboratory studies and cultures are performed to determine the microbe involved and its antibiotic sensitivities. Imaging with CT or ultrasound can diagnose up to 90% of hepatic abscess cases, with CT being the best modality of choice. In addition to diagnosis and location of the abscess, imaging should simultaneously be used to look for biliary disease or other intra-abdominal infections. MRI can follow if biliary disease or hepatic vein involvement is suspected; colonoscopy can follow to search for infection source in the gastrointestinal tract [13]. This rationale was used when obtaining CT imaging of the abdomen and pelvis, and CT-guided aspiration of the patient’s hepatic abscess.

The management of *S. intermedius* PLA can be unclear because no published guidelines are available. There is no consensus on appropriate treatment. Although intravenous antibiotics are considered first-line treatment. The duration of antibiotics has not been standardized either, but recommendations range from two to six weeks. Antibiotic sensitivities of the responsible microbe are used to guide antibiotic choice and treatment. Generally, when deciding the stopping point of therapy/determining treatment efficacy, a normal white blood cell count, apyrexia, normal CRP, and absence of pain are often used. Normalization or reversal of hepatic abscess on imaging often lags compared to clinical symptoms of the patient [9,13]. As referenced above, the 2012 publication concluded that the majority of abscesses were due to *Klebsiella* (45%) and *E. coli* (32%). In this study, drainage was used to treat abscesses ≥ 5 cm (considered large abscesses). The first line was percutaneous drainage, but surgical drainage was indicated if the abscess ruptured, contained multi-loculations, or the patient had biliary and/or intra-abdominal pathology as well. All 400 patients were started on intravenous antibiotics (third-generation cephalosporin or fluoroquinolone in combination with metronidazole and aminoglycoside). Of the patients, 32% improved on intravenous antibiotics alone and required no additional treatment. The remaining 68% required additional treatment; of this subgroup, 78.52% required percutaneous aspiration. The major complication of this study was the rupture of abscess into the pleural or peritoneal cavity (5% of patients). It is recommended that once the diagnosis of a hepatic abscess is made, diagnostic aspiration (either ultrasound or CT-guided) should be completed. If there are multiple abscesses, CT-guided aspiration is needed. During aspiration procedure, a drainage catheter is usually placed. The authors found that the patients who underwent aspiration only (no catheter placed) had a shorter hospital stay than patients who had a catheter placed (4.63 days versus seven days) [8]. Multivariate analyses demonstrated that the factors leading to poor prognosis (death or unhealed/uncured abscess) in patients with PLA were age and multiorgan dysfunction. Factors that were found to protect against poor prognosis were fever and length of hospital stay [7]. The presence of fever at presentation likely was a protective factor because it prompts physicians to look for the source of infection. Length of hospital stay may be a protective factor as patients’ symptoms, labs, and treatment course would be more closely monitored than in an outpatient setting. In a 2019 study of a New Zealand cohort, the most common organism causing PLA was *S. intermedius*, and the mean length of hospital stay was 10.3 days [11]. Patients with invasive *S. intermedius* infections have longer hospital stays and higher mortality rates than patients who had invasive infections with other SAG bacteria [14]. The case reported here required IV antibiotics and percutaneous aspiration with drainage, consistent with the majority of patients with PLA. However, the major complication (rupture into the pleural or peritoneal cavity) was not seen in our patient. Our patient had an extension of the abscess into pleural space, likely via fistula, without rupture. Also, his total hospital stay (first and second admissions) was 20 days, which is longer than the mean length of stay.

Compared to other cases of *S. intermedius* PLA, our patient’s presentation and disease course were unique, especially in the absence of documented risk factors for *S. intermedius* PLA. One case report documented the presentation and treatment of a 21-year-old immunocompetent patient with multiple PLAs and *S. intermedius* bacteremia, which resolved with ultrasound-guided percutaneous drainage and antibiotics. He had presented with RUQ pain [10]. There are a few notable differences between this patient’s case and our case report. First, the presentation is remarkably different from the patient we present here. Our patient did not present with abdominal pain, and therefore the location of infection could not be easily suspected to be of abdominal origin. If our patient had presented with RUQ abdominal pain, this would have given a clearer path to locating the abscess (as mentioned previously), as some of the most common presenting characteristics are upper abdominal pain (97%) and RUQ tenderness (95%). Also, though the 21-year-old was an immunocompetent individual, three months prior to the admission, the patient had a routine dental cleaning. This has been shown in the literature to predispose even immunocompetent patients to bacteremia from oral microbes such as *S. intermedius*. Our patient had not had a routine dental cleaning at the time of presentation; therefore, his bacteremia and PLA could not be attributed to this etiology. Another report detailed the course of a 71-year-old white male with a past medical history of hypertension, osteoarthritis, and benign prostatic hyperplasia, who had *S. anginosus* bacteremia with liver abscess secondary to a diverticular source. His diverticulosis/diverticulitis was found on imaging and was complicated by a colovesical fistula. The patient was discharged on day 24 of his hospital stay and the infection had completely resolved within three months of his discharge [6]. In comparison, our patient was a 39-year-old male without an extensive medical history or comorbidities. He also was found to have diverticulosis on a CT scan; however, it was not complicated by diverticulitis. It is possible that *S. intermedius* seeded our patient’s bloodstream via the colonic diverticula; however, no definitive source was found. In addition, our patient had further complications and required an additional hospital admission due to secondary pleural empyema and potential fistula formation.

This patient’s hospital course was complicated by persistent thrombocytosis and pleural empyema leading to rehospitalization two days following initial discharge. Thrombocytosis is defined as platelets elevated to or above 5 x 105/mL. Thrombocytosis is associated with an increased risk of longer hospital admissions, infections, sepsis, complications, and mortality. It is an independent predictor of mortality. Particularly, thrombocytosis is a clear biomarker for infections such as empyema, abscesses, and soft tissue infections [15]. The presence of pleural empyema secondary to *S. intermedius* PLA is a rare complication. Diaphragmatic extension of PLA has been documented; however, studies and case reports lack on the management of pleural empyema secondary to PLA extension across the diaphragm. The presence of pleural empyema secondary to PLA carries a negative impact on prognosis for patients [12] and it played a major part in the readmission of our patient. Our patient’s pleural empyema was managed by placement of a 12 French pigtail chest tube by interventional radiology, and IV ceftriaxone via PICC line course was extended. The chest drain was in place for six days and was removed in the outpatient clinic.

Particularly interesting are the potential reasons why our patient developed *S. intermedius* bacteremia, PLA, and subsequent pleural empyema if he did not have significant risk factors previously documented in the literature for *S. intermedius* infection.

One factor that we propose played a critical role in the development of bacteremia and abscess is the patient’s obesity. In a prospective cohort study published in 2020, obesity was shown to increase the risk of hospitalization for intra-abdominal infection, reproductive and urinary tract infection, skin and soft tissue infection, osteomyelitis, and necrotizing fasciitis in a dose-dependent manner (determined using accumulated hazard ratios (aHRs)). The greatest risk of obesity was for necrotizing fasciitis (aHR = 3.54), followed by skin and soft tissue infections (aHR = 2.46). Overweight and obese patients have been determined to have an increased risk of liver abscess as well, even after diabetes and blood glucose levels are adjusted for. The same study also determined that there was a U-shaped association curve of BMI with lower respiratory tract infections, all infections, and hospitalizations due to sepsis. Underweight patients and patients who were obese had a greater risk of infection than patients with BMI in the normal range [16].

Our patient’s BMI was 51.35 kg/m2, putting him into obesity class III and the category of extreme obesity (BMI of 40.0+). Thus, our patient at baseline had a greater risk of infection compared to a 39-year-old male with a normal BMI, particularly, soft tissue infections such as a PLA. The underlying mechanism of why obesity has increased risks for infection and poorer outcomes is somewhat still obscure. Through research on mouse models, it has been shown that adipocyte death can lead to liver injury and inflammation. Hepatic cells are sensitive to lipotoxicity, and their damage/inflammation leads to macrophage recruitment, further perpetuating liver injury. In a biochemical study published in 2019, researchers overexpressed human CD59 on mouse adipocytes and injected them with intermedilysin. Intermedilysin causes lysis of human CD59 expressing cells. When injected with intermedilysin, this caused the death of CD59 expressing adipocytes, leading to macrophage recruitment and elevated serum free fatty acids (due to lysis of adipocytes). They found that adipocyte death caused norepinephrine and epinephrine levels to increase, which lead to increased lipolysis, free fatty acids, and liver lipotoxicity. Liver CCR2+ macrophages were recruited and caused further liver damage [17]. Persistent inflammation and tissue damage allow the infection to ensue.

It could be argued that our patient developed *S. intermedius* bacteremia and PLA secondary to diverticulosis, as a diverticular disease was noted on the CT scan. Diverticular disease is not an uncommon finding; it is stated to be one of the most common pathologies noted in a routine colonoscopy. Almost one-third of patients over 45 years of age are estimated to have diverticular disease. Since mucosa of the colon is disrupted in diverticular disease, it can lead to the development of infection, hemorrhage, fistula formation, perforation, etc. In a 2015 article, where patients with diverticular disease were compared to controls (without diverticular disease), those with diverticular disease had an incidence of PLA 2.11 times higher than the controls. An adjusted hazard ratio of PLA was highest within the subgroup of patients less than 50 years of age (aHR = 4.03). The route of infection may be the spread of pathogens hematogenously from colonic mucosal defects in diverticular disease to the portal system and thus to the liver [18]. In the case presented, the patient was 39 years old (less than 50), with asymptomatic diverticulosis noted on the CT scan. However, no fat stranding or inflammation around diverticulosis, suggesting diverticulitis, was seen on imaging.

## Conclusions

This patient's case depicts the rare complication of empyema secondary to a PLA, likely due to communicating fistula between abscess and pleural cavity. The case adds to the medical literature, where a clear underrepresentation has been noted, as evidenced by our review of the literature and the lack of case reports documenting *S. intermedius* PLA complicated by pleural empyema and *S. intermedius* PLA in a patient without significant past medical history and no recent dental manipulation or cleaning. This case additionally demonstrates the impact of obesity on soft tissue and intra-abdominal infections, specifically liver abscesses, and *S. intermedius* intermedilysin lysis of adipocytes leading to lipotoxicity and damage of liver parenchyma. Additionally, this case highlights the possibility of PLAs and bacteremia in a patient without significant past medical history or common risk factors, and lack of typical physical exam findings such as the RUQ and abdominal tenderness.
